# Inferring the ecological niche of bat viruses closely related to SARS-CoV-2 using phylogeographic analyses of *Rhinolophus* species

**DOI:** 10.1038/s41598-021-93738-z

**Published:** 2021-07-12

**Authors:** Alexandre Hassanin, Vuong Tan Tu, Manon Curaudeau, Gabor Csorba

**Affiliations:** 1 Institut de Systématique, Évolution, Biodiversité (ISYEB), SU, MNHN, CNRS, EPHE, UA, Sorbonne Université, Paris, France; 2 Institute of Ecology and Biological Resources, Vietnam Academy of Science and Technology, No. 18, Hoang Quoc Viet Road, Cau Giay District, Hanoi, Vietnam; 3 Department of Zoology, Hungarian Natural History Museum, Baross u. 13, Budapest, H-1088 Hungary

**Keywords:** Population genetics, Animal migration, Biogeography, Ecological modelling, Evolutionary ecology, Infectious diseases, Viral infection, Evolutionary biology, Haplotypes

## Abstract

The Severe Acute Respiratory Syndrome coronavirus 2 (SARS-CoV-2) is the causal agent of the coronavirus disease 2019 (COVID-19) pandemic. To date, viruses closely related to SARS-CoV-2 have been reported in four bat species: *Rhinolophus acuminatus*, *Rhinolophus affinis*, *Rhinolophus malayanus*, and *Rhinolophus shameli*. Here, we analysed 343 sequences of the mitochondrial cytochrome c oxidase subunit 1 gene (*CO1*) from georeferenced bats of the four *Rhinolophus* species identified as reservoirs of viruses closely related to SARS-CoV-2. Haplotype networks were constructed in order to investigate patterns of genetic diversity among bat populations of Southeast Asia and China. No strong geographic structure was found for the four *Rhinolophus* species, suggesting high dispersal capacity. The ecological niche of bat viruses closely related to SARS-CoV-2 was predicted using the four localities in which bat viruses were recently discovered and the localities where bats showed the same *CO1* haplotypes than virus-positive bats. The ecological niche of bat viruses related to SARS-CoV was deduced from the localities where bat viruses were previously detected. The results show that the ecological niche of bat viruses related to SARS-CoV2 includes several regions of mainland Southeast Asia whereas the ecological niche of bat viruses related to SARS-CoV is mainly restricted to China. In agreement with these results, human populations in Laos, Vietnam, Cambodia, and Thailand appear to be much less affected by the COVID-19 pandemic than other countries of Southeast Asia. In the climatic transitional zone between the two ecological niches (southern Yunnan, northern Laos, northern Vietnam), genomic recombination between highly divergent viruses is more likely to occur. Considering the limited data and the risk of recombinant bat-CoVs emergence as the source of new pandemics in humans, the bat populations in these regions should be under surveillance.

## Introduction

The Severe Acute Respiratory Syndrome coronavirus 2 (SARS-CoV-2) emerged in December 2019 in Wuhan (China)^1^. After 17 months, the coronavirus disease 2019 (COVID-19) pandemic has affected more than 174 million of people around the world, claiming over 3.74 million lives^2^. The origin of SARS-CoV-2 remains enigmatic and many hypotheses have been advanced to explain the first animal-to-human transmission^3^.

**Figure 1:**
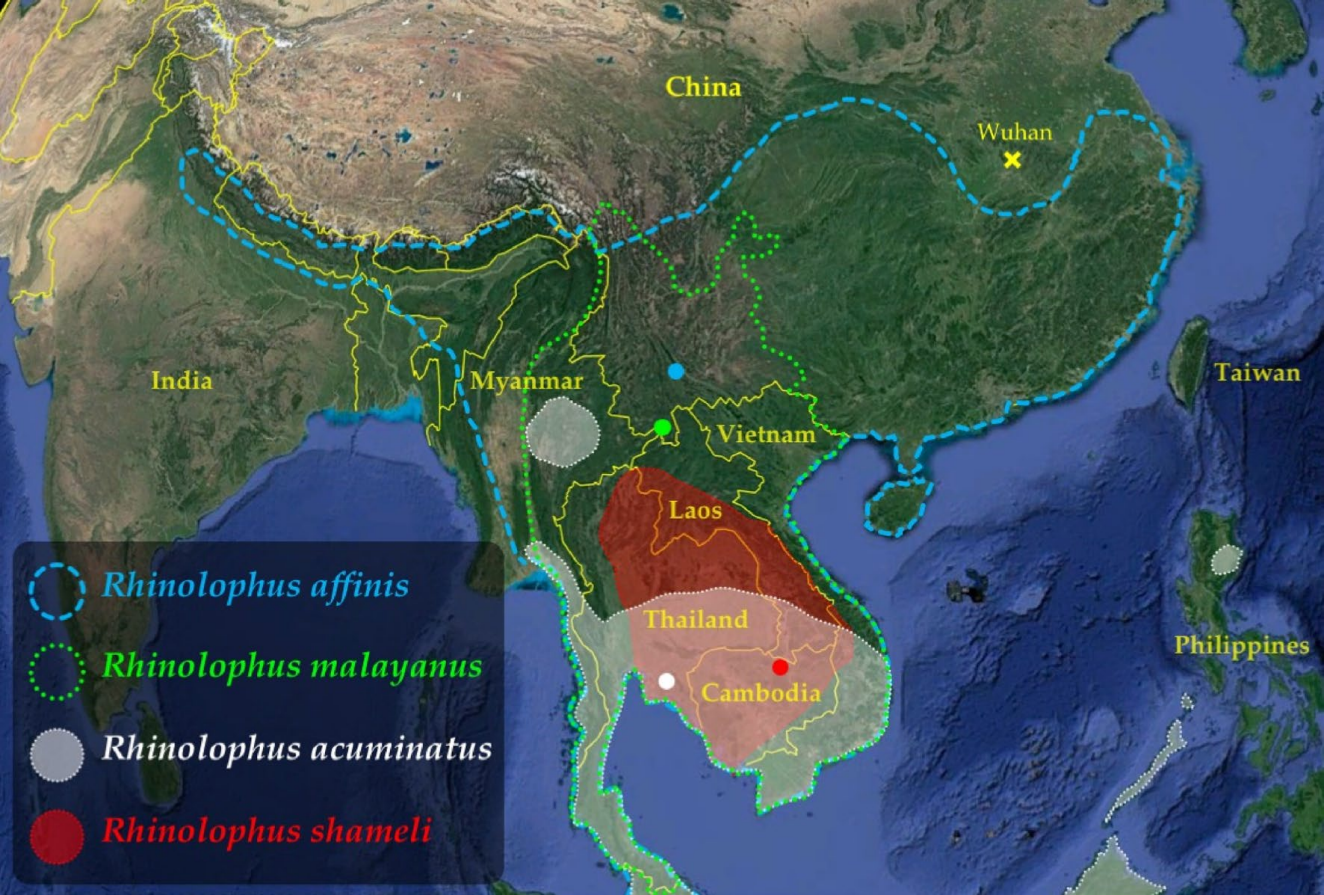
Geographic distribution of the four *Rhinolophus* species found positive for viruses closely related to SARS-CoV-2 in southern China and Southeast Asia. Map from Google Earth Pro (version 7.3.3.7786) US Dept of State Geographer © 2020 Google—Image Landsat/Copernicus—Data SIO, NOAA, U.S. Navy, NGA, GEBCO. For each of the four *Rhinolophus* species, the geographic distribution was extracted from the IUCN website^11^. The figure was drawn in Adobe Photoshop CS5 (version 12.0) and Microsoft PowerPoint (version 16.16.27). The coloured dots show the four geographic locations where bats found positive for SCoV2rCs were collected.

Within the family Coronaviridae, the subgenus *Sarbecovirus* includes two human viruses, SARS-CoV-2 and SARS-CoV (which was responsible for the SARS epidemic in 2002–2004)^4^. The genomes of these two viruses share only 80% of nucleotide identity, and whole-genome phylogenies have shown that they belong to two divergent lineages^1,5–7^, which we refer to herein as SARS-CoV related coronaviruses (SCoVrCs) and SARS-CoV-2 related coronaviruses (SCoV2rCs). Most SCoVrCs were discovered in bats collected in China after the SARS epidemic, and the great majority were found in horseshoe bat species of the genus *Rhinolophus* (family Rhinolophidae), suggesting that this taxon is the natural reservoir host of sarbecoviruses^8^. More recently, several viruses showing between 96.2 and 91.8% of genome identity with SARS-CoV-2 were identified from fecal samples of four horseshoe bat species: the RaTG13 virus (96.2%) was isolated from a *R. affinis* bat collected in Yunnan in 2013^1^; the RmYN02 virus (93.7%) was found in a *R. malayanus* bat sampled in Yunnan in 2019^5^; two variants of the same virus RshSTT200 (93.1%; other variant: RshSTT182) were detected in two *R. shameli* bats caught in northern Cambodia in 2010^6^; and five variants of the same virus RacCS203 (91.8%; other variants: RacCS224, RacCS253, RacCS264, and RacCS271) were sequenced from five *R. acuminatus* bats collected in eastern Thailand in 2020^7^. The bat species *R. acuminatus* and *R. shameli* are endemic to Southeast Asia whereas the two other bat species, *R. affinis* and *R. malayanus*, are distributed in both Southeast Asia and China (Fig. 1), suggesting that Southeast Asia is the main region where bat SCoV2rCs diversified. In addition, these recent data confirmed that the genus *Rhinolophus* is the natural reservoir host of all sarbecoviruses^3,8^. Note that this hypothesis was already corroborated by the discovery of two divergent sarbecovirus genomes (< 80% of genomic identity with SARS-CoV and SARS-CoV-2) in horseshoe bat species collected out of Asia: one in *Rhinolophus blasii* from Bulgaria (BM48-31)^9^ and another in an unidentified *Rhinolophus* species from Kenya (BtKY72)^10^.

**Figure 2:**
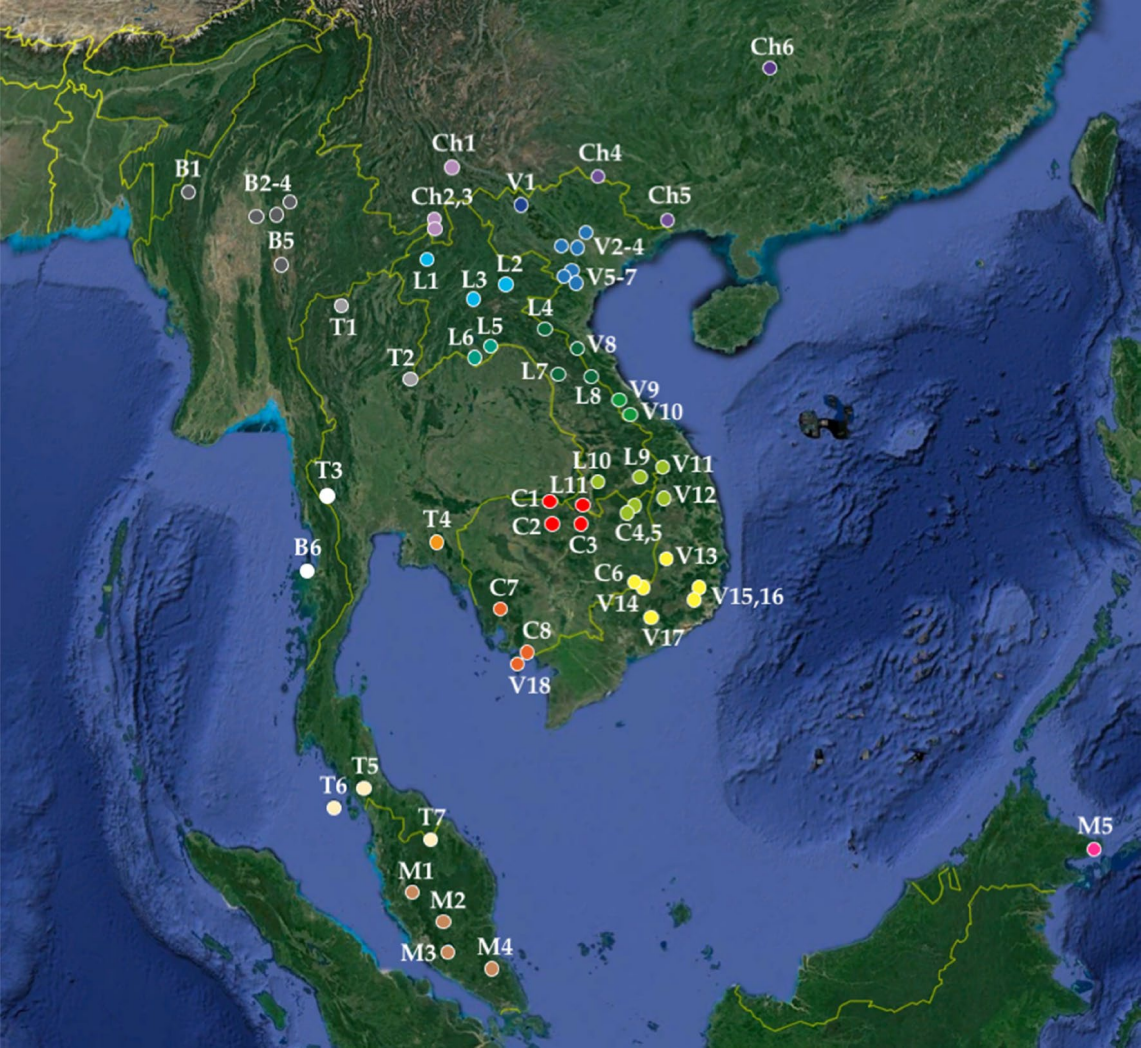
Geographic localities analysed in this study. The codes used for the countries are the following: B (Myanmar), C (Cambodia), Ch (China), I (Indonesia), L (Laos), M (Malaysia), T (Thailand), and V (Vietnam). Different regions were highlighted using colours. Map from Google Earth Pro (version 7.3.3.7786) US Dept of State Geographer © 2020 Google—Image Landsat/Copernicus—Data SIO, NOAA, U.S. Navy, NGA, GEBCO. The locality names and GPS coordinates are provided in online supplementary Table S1. The figure was drawn in Adobe Illustrator CS6 (version 16.0) and Microsoft PowerPoint (version 16.16.27).

Since SCoV2rCs have been circulating in horseshoe bats for many decades^12^, it is important to study population genetic structure of bats found positive for these sarbecoviruses in order to evidence their dispersal capacity in China and Southeast Asia. In this report, the phylogeography of the four species *R. acuminatus*, *R. affinis*, *R. malayanus* and *R. shameli* was therefore examined using the mitochondrial cytochrome c oxidase subunit 1 gene (CO1) from 343 georeferenced bats collected in 62 localities of Southeast Asia and China (Fig. 2). For each of the four species, haplotype networks were constructed to investigate geographic patterns of genetic diversity among bat populations. The results of these analyses were used to select specific location coordinates to predict the ecological niche of bat SCoV2rCs.

## Results and discussion

### Genetic analyses of *Rhinolophus* species identified as reservoirs of viruses closely related to SARS-CoV-2

**Figure 3:**
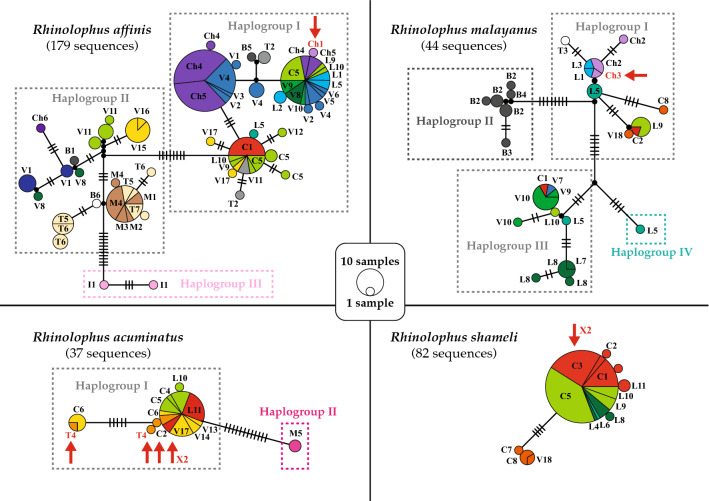
Haplotype networks based on *CO1* sequences of the four *Rhinolophus* species found positive for viruses closely related to SARS-CoV-2 (SCoV2rCs). The networks were constructed with the median joining method available in PopART 1.5^13^ and modified under Adobe Illustrator CS6 (version 16.0). The codes used for the countries are the following: B (Myanmar), C (Cambodia), Ch (China), I (Indonesia), L (Laos), M (Malaysia), T (Thailand), and V (Vietnam). Colours indicate the geographic origin of haplotypes according to Fig. 2 (see online supplementary Table S1). The circles indicate haplotypes separated by at least one mutation. The black lines on the branches show the number of mutations ≥ 2. Black circles represent missing haplotypes. Circle size is proportional to the number of haplotypes. Haplogroups separated by more than seven mutations (pairwise nucleotide distances > 1%) are highlighted by dotted lines. The red arrows show the positions of the nine bats found positive for SCoV2rCs.

Until now, SCoV2rCs have been found in four bat species of the genus *Rhinolophus*: *R. acuminatus*, *R. affinis*, *R. malayanus*, and *R. shameli*. The haplotype networks constructed using *CO1* sequences of these four species are shown in Fig. 3. A star-like genetic pattern, characterized by one dominant haplotype and several satellite haplotypes was found for the two bat species endemic to Southeast Asia, i.e. *R. acuminatus* and *R. shameli*.

In the network of *R. acuminatus*, the most common haplotype (named Rac1 in online supplementary Table S1) was found in northern Cambodia, southern Laos, eastern Thailand and southern Vietnam, indicating recent gene flow among these populations. Since a virus related to SARS-CoV-2 (91.8% of genome identity), named RacCS203, was detected in five *R. acuminatus* bats caught in eastern Thailand in June 2020^6^, the genetic pattern obtained for this species suggests that viruses closely related to RacCS203 may have circulated in most southern regions of mainland Southeast Asia. In contrast, *R. acuminatus* bats collected in Borneo (M5) showed a divergent haplotype (separated by 12 mutations; haplogroup II), suggesting that the South China Sea between mainland Southeast Asia and Borneo constitutes a barrier to gene flow. Isolated populations of *R. acuminatus* described in northern Myanmar, Indonesia (Java and Sumatra) and the Philippines^14^ should be further studied.

The network of *R. shameli* shows a typical star-like pattern, the most common haplotype (named Rsh1 in online supplementary Table S1) being detected in northern Cambodia and Laos. Since a virus related to SARS-CoV-2 (93.1% of genome identity), named RshSTT200, was recently discovered in two *R. shameli* bats collected in northern Cambodia in December 2010^7^, the genetic pattern obtained for this species suggests that viruses closely related to RshSTT200 may have circulated, at least in the zone between northern Cambodia and central Laos. The bats sampled south to the Tonle Sap lake (n = 4; southern Cambodia and Vietnamese island of Phu Quoc) were found to be genetically isolated from northern populations (four mutations). However, further sampling in the south is required to confirm this result, as it may reveal *CO1* sequences identical to the haplotypes detected in the north.

For the two species distributed in both China and Southeast Asia, i.e. *R. affinis* and *R. malayanus*, the genetic patterns are more complex with different haplogroups showing more than 1% of nucleotide divergence. In the network of *R. affinis*, there are three major haplogroups (named I, II and III in Fig. 3) separated by a minimum of seven mutations. The results are therefore in agreement with those previously published using *CO1* and D-loop mitochondrial sequences^15^. The *CO1* haplotypes detected in the localities sampled in southern China (ch1, ch4, ch5) are distantly related to the single haplotype available for central China (ch6), but they are also found in Laos, northern and central Vietnam, northern Thailand and northeastern Myanmar. This result suggests recent gene flow between populations from southern Yunnan and those from northern mainland Southeast Asia. Since a virus related to SARS-CoV-2 (96.2% of genome identity), named RaTG13, was detected in one *R. affinis* bat captured in southern Yunnan in 2013^1^, the genetic pattern obtained for this species suggests that viruses closely related to RaTG13 may have circulated in the zone comprising southern Yunnan and northern mainland Southeast Asia.

In the network of *R. malayanus*, there are four major haplogroups (named I, II, III and IV in Fig. 3) separated by a minimum of seven mutations. The *CO1* haplotypes detected in the localities sampled in southern China (ch2 and ch3) were also found in northern Laos (L1 and L3), suggesting recent gene flow between populations from these two countries. Since a virus related to SARS-CoV-2 (93.7% of genome identity), named RmYN02, was recently isolated from one *R. malayanus* bat collected in southern Yunnan in June 2019^5^, the genetic pattern obtained for this species suggests that viruses closely related to RmYN02 may have circulated, at least between southern Yunnan and northern Laos. In contrast, the bats sampled in Myanmar were found to be genetically isolated from other geographic populations (haplogroup II in Fig. 3).

### Two different ecological niches for bat viruses related to either SARS-CoV or SARS-CoV-2

In the wild, sarbecoviruses were generally detected after examining fecal samples collected on dozens of bats. For instance, two sarbecoviruses were found^7^ among the total 59 bats collected at the same cave entrance in northern Cambodia in 2010 (unpublished data). However, this does not mean necessarily that sarbecoviruses were absent in negative samples, as degradation of RNA molecules and very low viral concentrations may prevent the detection of RNA viruses. Despite these difficulties, full genomes of *Sarbecovirus* have been sequenced from a wide diversity of horseshoe bat species collected in Asia, Africa and Europe^5–10^. Therefore, there is no doubt that *Rhinolophus* species constitute the natural reservoir host of all sarbecoviruses^3,8^. The genus *Rhinolophus* currently includes between 92^11^ and 109^16^ insectivorous species that inhabit temperate and tropical regions of the Old World, with a higher biodiversity in Asia (63–68 out of the 92–109 described species) than in Africa (34–38 species), Europe (5 species) and Oceania (5 species). Although some *Rhinolophus* species are solitary, most of them are gregarious and live in large colonies or small groups generally in caves and hollow trees, but also in burrows, tunnels, abandonned mines, and old buildings^11,16^. However, they prefer large caves with total darkness, where temperatures are stable and less affected by diurnal and seasonal climatic variations. Importantly, all *Rhinolophus* species in which sarbecoviruses were detected in previous studies^1,5–9,17^ are cave species that form small groups or colonies (up to several hundreds)^11,18,19^.

**Figure 4:**
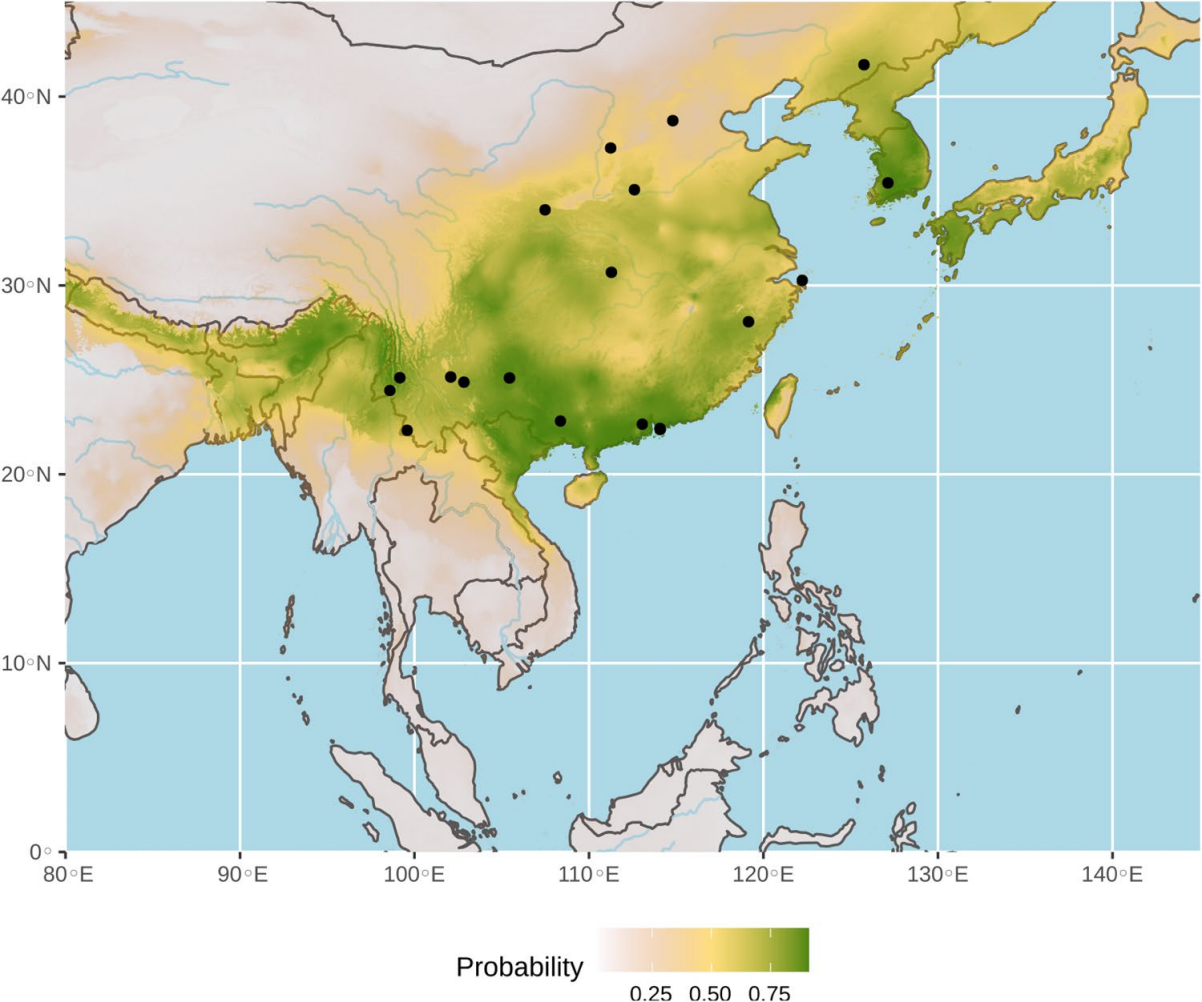
Ecological niche of bat viruses related to SARS-CoV (SCoVrCs). The geographic distribution of suitable environments was predicted using the Maxent algorithm in ENMTools (see “Methods” section for details). AUC = 0.81. Black circles indicate localities used to build the distribution model (see geographic coordinates in online supplementary Table S2).

In China, many SCoVrCs were previously detected in several horseshoe bat species, including *Rhinolophus sinicus*, *Rhinolophus ferrumequinum* (currently *R. nippon*)^16^, *Rhinolophus macrotis* (currently *R. episcopus*)^16^, *Rhinolophus pearsoni*, and *Rhinolophus pusillus*, and it has been shown that they circulate not only among conspecific bats from the same colony, but also between bat species inhabiting the same caves^17,20,21^. The ecological niche predicted for bat SCoVrCs using a data set of 19 points (see online supplementary Table S2) is shown in Fig. 4. The AUC was 0.81. The value was > 95% CI null-model’s AUCs (0.68), indicating that the model performs significantly better than a random model (see online supplementary Fig. S1). The highest probabilities of occurrence (highlighted in green in Fig. 4) were found in Nepal, Bhutan, Bangladesh, northeastern India, northern Myanmar, northern Vietnam, most regions of China south of the Yellow River, Taiwan, North and South Korea, and southern Japan.

In Southeast Asia and southern China, SCoV2rCs have currently been found in four *Rhinolophus* species (*R. acuminatus*, *R. affinis*, *R. malayanus* and R*. shameli*)^1,6–8^, but the greatest diversity of horseshoe bat species in mainland Southeast Asia (between 28 and 36 species)^11,16^ suggests that many sarbecoviruses will be discovered soon. Despite the limited data currently available on SCoV2rCs, several arguments support that bat intraspecific and interspecific transmissions also occur with SCoV2rCs. Firslty, recent genomic studies have revealed that SCoV2rCs circulate and evolve among horseshoe bats of the same colony, as five very similar genomes (nucleotide distances between 0.03% and 0.10%) were sequenced from five *R. acuminatus* bats collected from the same colony in eastern Thailand^6^, and as two genomes differing at only three nucleotide positions (distance = 0.01%) were sequenced from two *R. shameli* bats collected at the same cave entrance on the same night^7^. Secondly, the discovery of four viruses closely related to SARS-CoV-2 (between 96.2 and 91.8% of genome identity) in four different species of *Rhinolophus* is a strong evidence that interspecific transmission occurred several times in the past. As detailed in online supplementary Table S1, these species were collected together in several localities of Cambodia (three species in C1, C2, and C5; two species in C8), Laos (four species in L10; three species in L9; two species in L1, L5, L8, L11), and Vietnam (two speciess in V10, V9, V17, V18). These data corroborate previous studies suggesting that sarbecoviruses can be transmitted, at least occasionally, between *Rhinolophus* species sharing the same caves.

**Figure 5:**
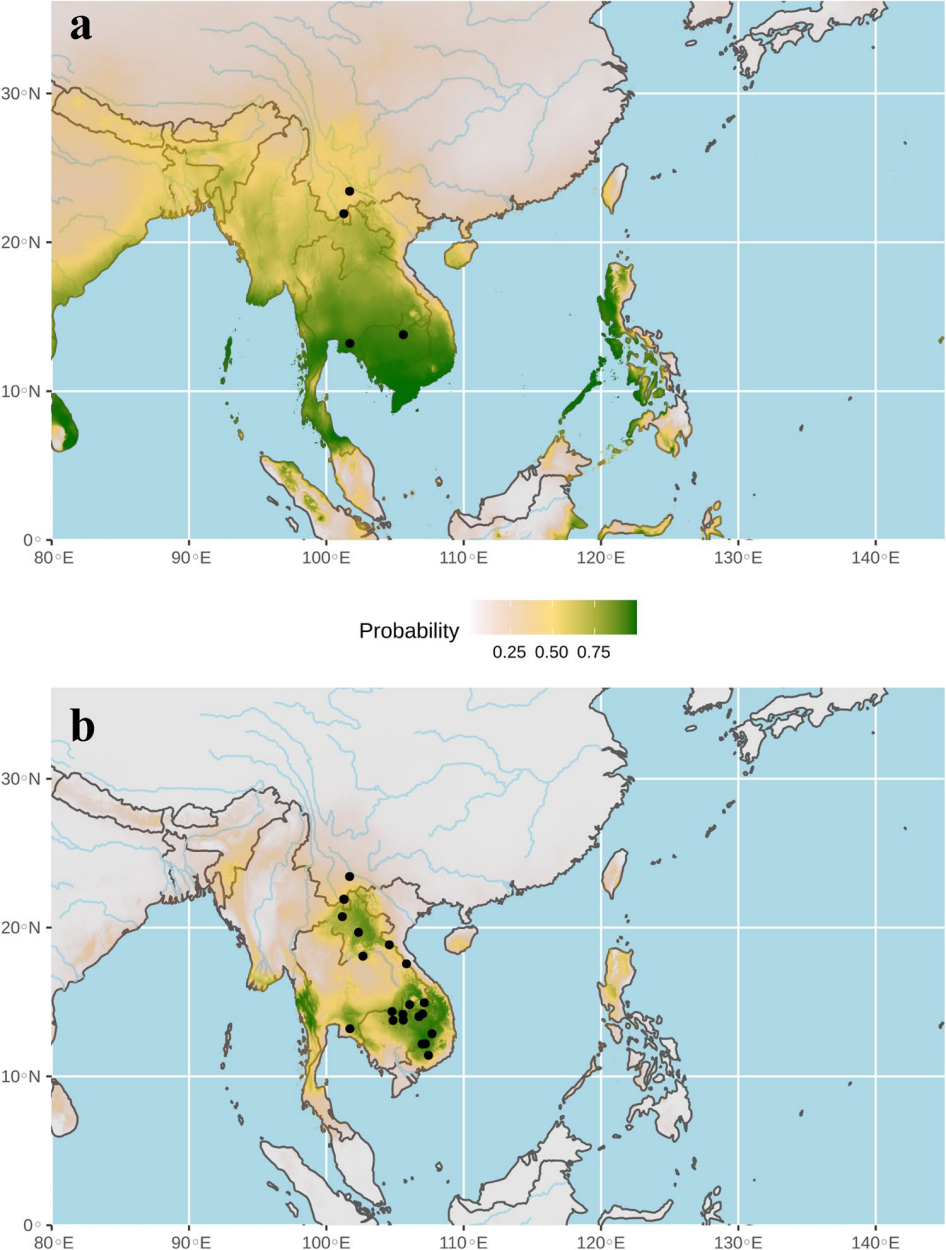
Ecological niches of bat viruses closely related to SARS-CoV-2 (SCoV2rCs) predicted using 4 points (**a**) (AUC = 0.58) and 21 points (**b**) (AUC = 0.96). The geographic distributions of suitable environments were predicted using the Maxent algorithm in ENMTools (see “Methods” section for details). Black circles indicate localities used to build the distribution model (see geographic coordinates in online supplementary Table S1).

The ecological niche of bat SCoV2rCs was firstly predicted using the four localities where bat viruses were previously detected^1,6–8^ (Fig. 5a). The highest probabilities of occurrence (highlighted in green in Fig. 5a) were found in Southeast Asia rather than in China. However, the AUC was only 0.58, and the value was < 95% CI null-model’s AUCs (0.74), indicating that the model was not statically supported at a significance level of 0.05 (see on line supplementary Fig. S2). As expected, these preliminary results confirmed that more than four records are needed to increase the accuracy of the distribution model^22^. For that reason, we used a genetic approach to determine geographic localities where bat SCoV2rCs are more likely to be found. The *CO1* sequences of the nine bats in which a SCoV2rC was detected are shown by red arrows in Fig. 3. For *R. affinis*, the *CO1* haplotype sequenced for the bat found positive for a SCoV2rC in southern Yunnan (site named Ch1 in Figs. 2, 3) was not found in other sampled localities. For the three other bat species found positive for SCoV2rCs, identical *CO1* sequences were detected in bats from 17 other geographic localities (see online supplementary Table S1). For *R. acuminatus*, the four *CO1* haplotypes sequenced for the bats found positive for SCoV2rCs in eastern Thailand (site named T4 in Figs. 2, 3) were also found in four localities in Cambodia (C2, C4, C5, and C6), two localities in southern Laos (L10 and L11), and three localities in southern Vietnam (V13, V14, and V17). The results indicate high connectivity among *R. acuminatus* populations from eastern Thailand, Cambodia, southern Laos and southern Vietnam. For *R. malayanus*, the *CO1* haplotype sequenced for the bat found positive for a SCoV2rC in southern Yunnan (site named Ch3 in Figs. 2, 3) was also found in another locality in southern Yunnan (Ch2) and two localities in northern Laos (L1 and L3). The results indicate high connectivity among *R. malayanus* populations from southern China and northern Laos. For *R. shameli*, the single *CO1* haplotype sequenced for the two bats found positive for SCoV2rCs in northern Cambodia (site named C3 in Figs. 2, 3) was also found in three other localities in Cambodia (C1, C2, and C5) and five localities in Laos (L4, L6, L8, L9, and L10). The results indicate high connectivity among *R. shameli* populations from Cambodia and Laos. Based on these genetic data, the ecological niche of bat SCoV2rCs was predicted using 21 records corresponding to the four localities where bat viruses were previously detected^1,6–8^ and the 17 localities where bats showed the same *CO1* haplotype than virus-positive bats (data set B: 21 points; see online supplemntary Table S1 for details). The AUC was 0.96. The value was > 95% CI null-model’s AUCs (0.81), indicating that the model performs significantly better than a random model (see online supplementary Fig. S3). The areas showing the highest probabilities of occurrence (highlighted in green in Fig. 5b) include four main geographic areas: (i) southern Yunnan, northern Laos and bordering regions in northern Thailand and northwestern Vietnam; (ii) southern Laos, southwestern Vietnam, and northeastern Cambodia; (iii) the Cardamom Mountains in southwestern Cambodia and the East region of Thailand; and (iv) the Dawna Range in central Thailand and southeastern Myanmar.

Our results show that bat SCoVrCs and SCoV2rCs have different ecological niches: that of SCoVrCs covers mainly China and several adjacent countries and extends to latitudes between 18° and 43°N, whereas that of SCoV2rCs covers northern mainland Southeast Asia and extends to latitudes between 10° and 24°N. Most *Rhinolophus* species involved in the ecological niche of SCoVrCs have to hibernate in winter when insect populations become significantly less abundant. This may be different for most *Rhinolophus* species involved in the ecological niche of SCoVrC2s. Since this ecological difference may be crucial for the dynamics of viral transmission among bat populations, it needs to be further studied through comparative field surveys in different regions of China and Southeast Asia. The ecological niches of SCoVrCs and SCoV2rCs slightly overlap in the zone including southern Yunnan, northern Laos, and northern Vietnam (Figs. 4, 5b). This zone corresponds to the northern edge of tropical monsoon climate^23^. Highly divergent sarbecoviruses of the two main lineages SCoVrCs and SCoV2rCs are expected to be found in sympatry in this area. This is confirmed by the discovery of both SCoVrCs and SCoV2rCs in horseshoe bats collected in southern Yunnan^1,6,21^. Collectively, these data suggest that genomic recombination between viruses of the two divergent lineages are more likely to occur in bats roosting, at least seasonally, in the caves of these regions. Since highly recombinant viruses can threaten the benefit of vaccination campaigns, southern Yunnan, northern Laos, and northern Vietnam should be the targets of closer surveillance.

### Mainland Southeast Asia is the cradle of diversification of bat SCoV2rCs

**Figure 6:**
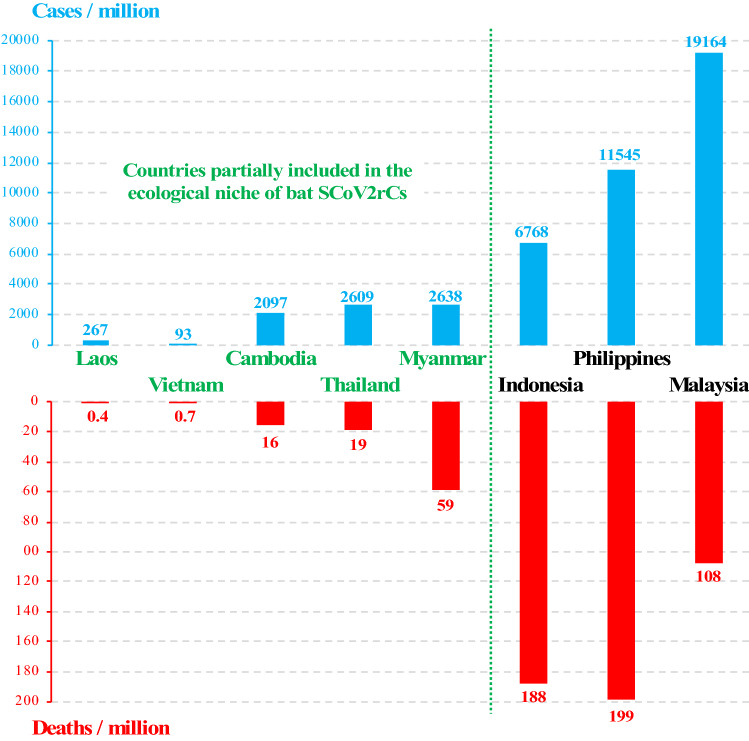
Number of COVID-19 patients per million inhabitants (in blue) and deaths per million inhabitants (in red) for the different countries of Southeast Asia. Data extracted from the Worldometers website^2^ on June 08, 2021. The figure was drawn in Microsoft Excel and PowerPoint (version 16.16.27).

Chinese researchers have actively sought sarbecoviruses in all Chinese provinces after the 2002–2004 SARS outbreak. They found many bat SCoVrCs^16,20,21^ but only two SCoV2rCs^1,5^ and both of them were discovered in southern Yunnan, the Chinese province bordering Southeast Asia. The ecological niches predicted herein for bat sarbecoviruses suggest that SCoVrCs are dominant in China (Fig. 4) while SCoV2rCs are present mostly in Southeast Asia (Fig. 5). This means that viruses similar to SARS-CoV-2 have been circulating for several decades throughout Southeast Asia, and that different species of bats have exchanged these viruses in the caves they inhabit. The data available on human cases and deaths caused by the COVID-19 pandemic^2^ indirectly support the hypothesis that the cradle of diversification of bat SCoV2rCs is mainland Southeast Asia, and in particular the areas highlighted in green in Fig. 5b. Indeed, human populations in Cambodia, Laos, Thailand, and Vietnam appear to be much less affected by the COVID-19 pandemic than other countries of the region, such as Indonesia, Malaysia, Myanmar, and the Philippines (Fig. 6). This suggests that some human populations of Cambodia, Laos, Thailand, and Vietnam, in particular rural populations living in contact with wild animals for several generations, have a better immunity against SCoV2rCs because they have been regularly contaminated by bats and/or infected secondary hosts such as pangolins.

### Pangolins contaminated by bats in Southeast Asia

Apart from bats, the Sunda pangolin (*Manis javanica*) and Chinese pangolin (*Manis pentadactyla*) are the only wild animals in which viruses related to SARS-CoV-2 have been found so far. However, these discoveries were made in a rather special context, that of pangolin trafficking. Several sick pangolins were seized by Chinese customs in Yunnan province in 2017 (unpublished data), in Guangxi province in 2017–2018^24^ and in Guangdong province in 2019^25^. Even if the viruses sequenced in pangolins are not that close to SARS-CoV-2 (one was 85% identical and the other 90%), they indicate that at least two sarbecoviruses could have been imported into China well before the emergence of COVID-19 epidemic. Indeed, it has been shown that Sunda pangolins collected from different Southeast Asian regions have contaminated each other while in captivity on Chinese territory^3^. It has been estimated that 43% of seized pangolins were infected by at least one SARS-CoV-2-like virus^3^. Such a high level of viral prevalence and the symptoms of acute interstitial pneumonia detected in most dead pangolins^24^ indicate that captive pangolins are highly permissive to infection by SARS-CoV-2-like viruses. The question remained on how the Sunda pangolins became infected initially. Could it have been in their natural Southeast Asian environment, before being captured? The discovery of two new viruses close to SARS-CoV-2 in bats from Cambodia and Thailand^7,8^ supports this hypothesis, as *Rhinolophus* bats and pangolins can meet, at least occasionally, in forests of Southeast Asia, possibly in caves, tree hollows or burrows. Further substantiating this hypothesis, the geographic distribution of *Manis javanica*^26^ overlaps the ecological niche here predicted for bat SCoV2rCs (Fig. 5), and SARS-CoV-2 neutralizing antibodies have been recently detected in one of the ten pangolin sera sampled from February to July 2020 from three wildlife checkpoint stations in Thailand^6^. Collectively, these data strengthen the hypothesis that pangolin trafficking is responsible for multiple exports of viruses related to SARS-CoV-2 to China^3^.

## Methods

### DNA extraction and sequencing

Tissue samples (n = 144) of morphologically identified bats of *R. acuminatus* (n = 10), *R. affinis* (n = 57), *R. malayanus* (n = 14), and *R. shameli* (n = 63) were analysed for this study. These bats were captured with mist nets and harp-traps during several field surveys in Cambodia (2010), Laos (2007) and Vietnam (2011) to promote bat conservation. In 2007, the Muséum national d’Histoire naturelle (MNHN, Paris, France) was mandated by UNESCO and the World Heritage House of Luang Prabang to conduct a mammal survey in northern Laos. In 2010, the MNHN was mandated by UNESCO and the National Authority of Preah Vihear to conduct a mammal survey in northern Cambodia. Field surveys in Vietnam were authorized by the Forest Protection Department of the Ministry of Agriculture and Rural Development, the local manager boards of different protected areas (see online supplementary Table S1), and the Institute of Ecology and Biological Resources (IEBR, Hanoi, Vietnam). The animals were handled according to guidelines and recommendations of the American Society of Mammologists^27^. They were measured, photographed and identified by the authors (AH, GS and VTT). Tissue samples were taken from the chest muscles of voucher specimens or from the patagium (biopsy punches; 2 mm diameter) of released bats. Samples were preserved in 95% ethanol.

Total DNA was extracted using QIAGEN DNeasy Tissue Kit (Qiagen, Germany) in accordance with the manufacturer’s instructions. The barcode fragment of the *CO1* gene (657 bp) was amplified and sequenced using the primers UTyr and C1L705^28^. PCR amplifications of the *CO1* gene were performed as previously published^29^. PCR products were purified using ExoSAP Kit (GE Healthcare, UK) and sequenced using the Sanger method on an ABI 3730 automatic sequencer at the Centre National de Séquençage (Genoscope) in Evry (France). Haplotypes were assembled with forward and reverse eletcropherograms using Sequencher 5.1 (Gene Codes Corporation, Ann Arbor, MI, USA). No gaps and stop codons were found in the *CO1* sequences after translation into amino-acids. Sequences generated for this study were deposited in the GenBank database (accession numbers MW712891-MW713034) (see online supplementary Table S1).

### Analyses of CO1 sequences

Our sequences were aligned with 199 additional *CO1* sequences downloaded from GenBank. Note that the *CO1* sequences of seven bats found positive for viruses closely related to SARS-CoV-2^1,5,6^ were assembled on Geneious Prime 2020.0.3 (Biomatters Ltd., Auckland, New Zealand) by mapping available SRA data to a *CO1* reference. Sequences were aligned using AliView 1.22^30^. Our final *CO1* alignments contain 37 sequences for *R. acuminatus*, 44 sequences for *R. malayanus*, 82 sequences for *R. shameli*, and 180 sequences for *R. affinis*. These four alignments were analysed in PopART 1.5^13^ to construct haplotype networks using the median joining method with equal weights for all mutations. The 62 localities where bats were sampled are shown in the map of Fig. 2 and their geographic coordinates are detailed in online supplementary Table S1.

### Prediction of ecological niches

For bat SCoVrCs, the ecological niche was inferred using GPS data collected for viruses published during the last two decades. The list of the 19 available geographic records is provided in online supplementary Table S2. For bat SCoV2rCs, the ecological niche was initially predicted using the four geographic localities where viruses were previously detected^1,5–7^: two in Yunnan, one in northern Cambodia, and one in eastern Thailand (data set A). However, the use of only four records is questionable since Van Proosdij et al.^22^ have estimated that a minimum of 13 records is required to develop accurate distribution models for widespread taxa. For that reason, we used a genetic approach to increase the number of geographic records. Since the detection of identical *CO1* sequences in different bat populations is indicative of recent dispersal events of females, we also selected the 17 geographic records where bats showed the same *CO1* haplotypes than virus-positive bats (data set B: 21 points; see online supplementary Table S1).

For each of the three data sets (bat SCoVrCs; data sets A and B for bat SCoV2rCs), the 19 bioclimatic variables available in the WorldClim database^31^ were studied for an area corresponding to the minimum and maximum latitudes and longitudes of the selected points (19 points for bat SCoVrCs; 4 and 21 points, respectively for the SCoV2rCs data sets A and B) and the caret R package^32^ was used to determine the least correlated variables (|r|< 0.7)^33^. For bat SCoVrCs, the following five predictor bioclimatic variables were retained: Bio3 (isothermality), Bio4 (temperature seasonality), Bio5 (maximum temperature of the warmest month), Bio15 (precipitation seasonality), and Bio18 (precipitation of the warmest quarter). For data set A, the following seven predictor bioclimatic variables were retained: Bio3, Bio7 (temperature annual range), Bio10 (mean temperature of the warmest quarter), Bio13 (precipitation of the wettest month), Bio14 (precipitation of driest month), Bio15, and Bio18. For data set B, the following seven predictor bioclimatic variables were selected: Bio2 (mean diurnal range), Bio3, Bio7, Bio10, Bio13, Bio15, Bio17 (precipitation of the driest quarter), and Bio18. Ecological niche modelling was performed with the MaxEnt algorithm using ENMTools in R^34^. The MaxEnt approach was chosen for its ability to work with presence-only data sets and to produce results with a low sample size^35^. The area under the curve (AUC) of the receiver operating characteristic plot was used as a first measure of model accuracy, a value of 0.5 indicating model accuracy not better than random, and a value of 1 indicating perfect model fit^36,37^. To test for sampling bias, the distribution model using all selected localities was tested against a null model developed by 1000 times drawing an equal number of random points from the entire study area^37^. The position of the AUC value was tested against the 95% confidence interval (CI) of the 1000 AUC values of the null-models. If the AUC value is ≥ 95% CI null-model’s AUCs, the model is considered performing significantly better than a random model^37^.

### Ethical statement

Ethical review and approval were not available for our study because the field missions were carried out between 2004 and 2011, i.e., before the creation of the ethical committee at the Muséum national d’Histoire naturelle. However, the field studies were carried out in compliance with the ARRIVE guidelines.

## Supplementary Information

Supplementary Information.

## Data Availability

DNA sequences generated for this study were deposited in the GenBank database (Accession Numbers MW712891-MW713034).
