# Supplementary material for: Inferring the ecological niche of bat viruses closely related to SARS-CoV-2 using phylogeographic analyses of Rhinolophus species
Source: Sci Rep. 2021 Jul 12;11:14276. doi: 10.1038/s41598-021-93738-z (PMC8275577; doi:10.1038/s41598-021-93738-z)

Supplementary Information

Supplementary Table S1. Cytochrome c oxidase subunit 1 (CO1) haplotypes of *Rhinolophus* species analysed in this study

Supplementary Table S2. Geographic records used to infer the ecological niche of bat viruses related to SARS-CoV.

Supplementary Figure S1. Model performance in geographic space on training data.
Ecological niche of bat viruses related to SARS-CoV (SCoVrCs).

Supplementary Figure S2. Model performance in geographic space on training data.
Ecological niches of bat viruses closely related to SARS-CoV-2 (SCoV2rCs) predicted using 4 points (data set A).

Supplementary Figure S3. Model performance in geographic space on training data.
Ecological niches of bat viruses closely related to SARS-CoV-2 (SCoV2rCs) predicted using 21 points (data set B).

Table S1. Cytochrome c oxidase subunit 1 (*CO1*) haplotypes of *Rhinolophus* species analysed in this study.

Species	GenBank accession numbers	Haplotypes	Country	Location	Code	Latitude	Longitude	References	EN datasets
R. acuminatus	MW712895, MW712896	Rac1	Cambodia	Krong Preah Vihear	C2	13.7641	104.8592	This study	20 points, 30 points
R. acuminatus	MW712894	Rac1	Cambodia	near Kok Lak, Rattanakiri	C4	14.0186	106.7484	This study	20 points, 30 points
R. acuminatus	MW712891-MW712893	Rac1	Cambodia	Virachey	C5	14.2032	107.0050	This study	20 points, 30 points
R. acuminatus	MW712897	Rac1	Cambodia	Mondolkiri	C6	12.1803	106.9819	This study	20 points, 30 points
R. acuminatus	MW712898- MW712900	Rac2	Cambodia	Mondolkiri	C6	12.1803	106.9819	This study	20 points, 30 points
R. acuminatus	HM541312, HM541313, HM541317, HM541318	Rac1	Laos	Dong Hua Sao	L10	14.8220	106.0600	[1]	20 points, 30 points
R. acuminatus	HM541319, HM541320, HM541321, HM541322, HM541323, HM541324, HM541314	Rac1	Laos	Dong Kanthung	L11	14.1750	105.5750	[1]	20 points, 30 points
R. acuminatus	HM541315	Rac6	Laos	Dong Hua Sao	L10	14.8220	106.0600	[1]	30 points
R. acuminatus	HM541310, HM541311	Rac3	Malaysia	Tabin	M5	5.0830	118.6670	[1]	
R. acuminatus	SRR13380249, SRR13380251	Rac1	Thailand	Khao Ang Rue Nai	T4	13.2067	101.7119	[2]	4 points, 20 points, 30 points
R. acuminatus	SRR13380250	Rac2	Thailand	Khao Ang Rue Nai	T4	13.2067	101.7119	[2]	4 points, 20 points, 30 points
R. acuminatus	SRR13380247	Rac4	Thailand	Khao Ang Rue Nai	T4	13.2067	101.7119	[2]	4 points, 20 points, 30 points
R. acuminatus	SRR13380248	Rac5	Thailand	Khao Ang Rue Nai	T4	13.2067	101.7119	[2]	4 points, 20 points, 30 points
R. acuminatus	HM541316	Rac1	Vietnam	Yok Don NP	V13	12.8670	107.7000	[1]	20 points, 30 points
R. acuminatus	HM914924, HM914925	Rac1	Vietnam	Bu Gia Map	V14	12.1940	107.2060	[2]	
R. acuminatus	HM541325, HM914901, HM914902, HM914913, HM914914	Rac1	Vietnam	Cat Tien	V17	11.4170	107.4330	[1]	20 points, 30 points
R. affinis	MW712901- MW712909	Ra1	Cambodia	Preah Vihear Temple	C1	14.3594	104.7859	This study	
R. affinis	MW712910- MW712912	Ra1	Cambodia	Virachey	C5	14.2032	107.0050	This study	
R. affinis	MW712913, MW712914	Ra2	Cambodia	Virachey	C5	14.2032	107.0050	This study	
R. affinis	MW712915- MW712921	Ra3	Cambodia	Virachey	C5	14.2032	107.0050	This study	30 points
R. affinis	MW712922	Ra4	Cambodia	Virachey	C5	14.2032	107.0050	This study	
R. affinis	HM541383, HM541387	Ra3	China	Guangxi, Jing Xin County Provincial Nature Reserve	Ch4	23.1170	105.9670	[1]	30 points
R. affinis	HM541347, HM541348, HM541353	Ra3	China	Guangxi, Shiwandashan National Reserve	Ch5	21.8460	107.8890	[1]	30 points
R. affinis	SRR11085797	Ra5	China	Moijang	Ch1	23.4319	101.6924	[3]	4 points, 20 points, 30 points
R. affinis	HM541342, HM541343-6, HM541377-82, HM541384-6, HM541388-90, HM541392-4	Ra6	China	Guangxi, Jing Xin County Provincial Nature Reserve	Ch4	23.1170	105.9670	[1]	
R. affinis	HM541349, HM541350-2, HM541354-9, HM541368-76	Ra6	China	Guangxi, Shiwandashan National Reserve	Ch5	21.8460	107.8890	[1]	
R. affinis	HM541391	Ra7	China	Guangxi, Jing Xin County Provincial Nature Reserve	Ch4	23.1170	105.9670	[1]	
R. affinis	JF444035	Ra8	China	Shuhuangshan Reserve	Ch6	26.4167	111.0330	unpublished	
R. affinis	KP192694	Ra16	Indonesia	Sumatra	I1	-4.5597	105.4068	[4]	
R. affinis	KP192693	Ra17	Indonesia	Sumatra	I1	-4.5597	105.4068	[4]	
R. affinis	HM541335	Ra1	Laos	Dong Hua Sao	L10	14.8220	106.0600	[1]	
R. affinis	HM541336, HM541341	Ra10	Laos	Xiangkhoang, Nam Et NBCA	L2	20.0730	103.4330	[1]	
R. affinis	HM541339, HM541340	Ra3	Laos	LouangNamtha	L1	20.7360	101.1670	[1]	30 points

R. affinis	HM541334	Ra3	Laos	Dong Hua Sao	L10	14.8220	106.0600	[1]	30 points
R. affinis	HM541366, HM541367	Ra3	Laos	TakLeuk	L5	18.3830	103.0670	[1]	30 points
R. affinis	HM541333	Ra3	Laos	Attapeu	L9	14.9500	107.1330	[1]	30 points
R. affinis	HM541365	Ra9	Laos	TakLeuk	L5	18.3830	103.0670	[1]	
R. affinis	HM541337, HM541338	Ra11	Malaysia	Cameron Highlands	M1	4.4600	101.3800	[1]	
R. affinis	HM541330	Ra11	Malaysia	Kuala Lompat	M2	3.7170	102.1670	[1]	
R. affinis	HM541331, HM541332	Ra11	Malaysia	Pasoh Forest	M3	2.9670	102.3000	[1]	
R. affinis	HM541411, HM541412, HM541413, HM541407-10	Ra11	Malaysia	Endau Rompin	M4	2.5330	103.4000	[1]	
R. affinis	HM541414	Ra12	Malaysia	Endau Rompin	M4	2.5330	103.4000	[1]	
R. affinis	MK410386	Ra13	Myanmar	Chin	B1	22.8065	93.6998	[5]	
R. affinis	HM541326	Ra14	Myanmar	Nyaungshwe	B5	20.6630	96.9670	[1]	
R. affinis	KP192682	Ra15	Myanmar	Kadan	B6	12.4739	98.4032	[4]	
R. affinis	KY034102, KY034135	Ra1	Thailand	Loei	T2	17.5050	100.9380	unpublished	
R. affinis	KP192677, KP192678	Ra11	Thailand	Khuan Kalong	T5	7.0129	100.0210	[4]	
R. affinis	KY034109	Ra11	Thailand	Songkla	T6	6.9940	100.1490	unpublished	
R. affinis	KP192673, KP192675, KP192676	Ra11	Thailand	Hala Bala	T7	5.7983	101.8250	[4]	
R. affinis	KY034138	Ra18	Thailand	Loei	T2	17.5050	100.9380	unpublished	
R. affinis	KP192689, KP192690, KP192691	Ra19	Thailand	Adang	T6	6.5146	99.3173	[4]	
R. affinis	KY034134	Ra20	Thailand	Songkla	T6	6.9940	100.1490	unpublished	
R. affinis	KP192687, KP192688	Ra21	Thailand	Chieng Mai	T1	19.5093	98.8326	[4]	
R. affinis	KP192679, KP192680, KP192681	Ra22	Thailand	KhuanKalong	T5	7.0129	100.0210	[4]	
R. affinis	KP192692, KY034116, KY034119	Ra22	Thailand	Adang	T6	6.5146	99.3173	[4]	
R. affinis	KP192674	Ra23	Thailand	Hala Bala	T7	5.7983	101.8250	[4]	
R. affinis	HM541360	Ra1	Vietnam	Ngoc Linh, Quang Nam	V11	15.2088	107.7395	[1]	
R. affinis	HM541363	Ra1	Vietnam	Cat Tien	V17	11.4170	107.4330	[1]	
R. affinis	MW712923	Ra1	Vietnam	Huong Hoa	V9	16.9370	106.5810	This study	
R. affinis	MW712924, MW712925, HM541399, HQ580330	Ra24	Vietnam	Tram Ton, Sapa	V1	22.3278	103.7792	This study, [1], unpublished	
R. affinis	MW712926	Ra25	Vietnam	Hanoi, Ba Vi National park	V4	21.0830	105.3730	This study	
R. affinis	MW712927-MW712929	Ra26	Vietnam	Hanoi, Ba Vi National park	V4	21.0830	105.3730	This study	
R. affinis	MW712930, HM541402	Ra27	Vietnam	Ngoc Linh, Kon Tum	V11	15.2088	107.7395	This study, [1]	
R. affinis	MW712931	Ra28	Vietnam	Ngoc Linh, Kon Tum	V11	15.2088	107.7395	This study	
R. affinis	MW712932	Ra29	Vietnam	Kon Tum, Chu Mom Ray	V12	14.4250	107.7860	This study	
R. affinis	MW712933	Ra3	Vietnam	Dakrong	V10	16.6000	106.8667	This study	30 points
R. affinis	KP192684	Ra3	Vietnam	Vinh Phuc, Tam Dao National Park	V2	21.5075	105.6082	[6]	30 points
R. affinis	MW712934- MW712936	Ra3	Vietnam	Hanoi, Ba Vi National park	V4	21.0830	105.3730	This study	30 points
R. affinis	MW712937	Ra3	Vietnam	Thanh Hoa, Ba Thuoc	V5	20.4600	105.2520	This study	30 points
R. affinis	MW712938	Ra3	Vietnam	Thanh Hoa, Pu Luong Nature Reserve	V6	20.3050	105.0120	This study	30 points
R. affinis	MW712939-MW712942	Ra3	Vietnam	Vu Quang	V8	18.3200	105.4360	This study	30 points

R. affinis	MW712943-MW712945	Ra3	Vietnam	Huong Hoa	V9	16.9370	106.5810	This study	30 points
R. affinis	HM541398, HQ580331	Ra30	Vietnam	Sapa, Hoang Lien NP	V1	22.3278	103.7792	[1], unpublished	
R. affinis	HM541364	Ra31	Vietnam	Lao Cai, Ta Phinh	V1	22.3278	103.7792	[1]	
R. affinis	KP192683	Ra32	Vietnam	Vinh Phuc, Tam Dao National Park	V2	21.5075	105.6082	[4]	
R. affinis	MW712946, HM541395, HM541401	Ra33	Vietnam	Ngoc Linh, Quang Nam	V11	15.2088	107.7395	[1]	
R. affinis	GU684791, GU684792-4, GU684798, GU684801, HM541327	Ra34	Vietnam	Bi Doup-Nui Ba	V15	12.1790	108.6790	[1], unpublished	
R. affinis	HM541396	Ra35	Vietnam	Xuan Truong	V16	11.8670	108.5500	[1]	
R. affinis	HM541361	Ra36	Vietnam	Cat Tien	V17	11.4170	107.4330	[1]	
R. affinis	HM541362	Ra37	Vietnam	Cat Tien	V17	11.4170	107.4330	[1]	
R. affinis	HM541328	Ra38	Vietnam	Vu Quang	V8	18.3200	105.4360	[1]	
R. affinis	HM541329	Ra39	Vietnam	Vu Quang	V8	18.3200	105.4360	[1]	
R. affinis	KP192685, KP192686	Ra6	Vietnam	Vinh Phuc, Tam Dao National Park	V2	21.5075	105.6082	[4]	
R. affinis	MW712947	Ra6	Vietnam	Phu Tho, Xuan Son	V3	21.1386	104.9399	This study	
R. affinis	MW712948-MW712957	Ra6	Vietnam	Hanoi, Ba Vi National park	V4	21.0830	105.3730	This study	
R. malayanus	MW712958	Rm1	Cambodia	Preah Vihear Temple	C1	14.3594	104.7859	This study	
R. malayanus	MW712959	Rm2	Cambodia	Krong Preah Vihear	C2	13.7641	104.8592	This study	
R. malayanus	MW712960	Rm3	Cambodia	Kampot	C8	10.5597	104.2822	This study	
R. malayanus	MK900704, MK900705	Rm4	China	Yunnan	Ch2	21.8896	101.2979	[7]	20 points, 30 points
R. malayanus	SRR12432009	Rm4	China	Mengla County	Ch3	21.9189	101.2716	[8]	4 points, 20 points, 30 points
R. malayanus	MK900706	Rm5	China	Yunnan	Ch2	21.8896	101.2979	[7]	
R. malayanus	HM541621	Rm10	Laos	Hin Nam No	L8	17.5670	105.8330	[1]	
R. malayanus	HM541622	Rm11	Laos	Hin Nam No	L8	17.5670	105.8330	[1]	
R. malayanus	HM541610	Rm12	Laos	Dong Hua Sao	L10	14.8220	106.0600	[1]	
R. malayanus	HM541606, HM541607, HM541608, HM541609	Rm2	Laos	Attapeu	L9	14.9500	107.1330	[1]	
R. malayanus	HM541613	Rm4	Laos	LouangNamtha	L1	20.7360	101.1670	[1]	20 points, 30 points
R. malayanus	MW712961	Rm4	Laos	Nam Khan	L3	19.6823	102.3377	This study	20 points, 30 points
R. malayanus	HM541614, HM541615, HM541616	Rm6	Laos	TakLeuk	L5	18.3830	103.0670	[1]	30 points
R. malayanus	HM541619	Rm7	Laos	TakLeuk	L5	18.3830	103.0670	[1]	
R. malayanus	HM541620	Rm8	Laos	TakLeuk	L5	18.3830	103.0670	[1]	
R. malayanus	HM541618	Rm9	Laos	Ban Pomkhoun-Phonsong	L7	17.6330	104.9500	[1]	
R. malayanus	HM541624, HM541617, HM541623	Rm9	Laos	Hin Nam No	L8	17.5670	105.8330	[1]	
R. malayanus	KX099327, KX099330	Rm13	Myanmar	NA	B2	22.0476	95.9993	[9]	
R. malayanus	HM541612	Rm13	Myanmar	NA	B3	22.1000	96.6200	[1]	
R. malayanus	KX099328, KX099329	Rm14	Myanmar	NA	B2	22.0476	95.9993	[9]	
R. malayanus	KX099331	Rm15	Myanmar	NA	B2	22.0476	95.9993	[9]	
R. malayanus	KX099332	Rm16	Myanmar	NA	B2	22.0476	95.9993	[9]	
R. malayanus	HM541611	Rm17	Myanmar	NA	B4	22.4690	96.9930	[1]	
R. malayanus	MW712962	Rm19	Thailand	Kanchanaburi	T3	14.4500	98.8500	This study	
R. malayanus	MW712966-MW712971	Rm1	Vietnam	Dakrong	V10	16.6000	106.8667	This study	
R. malayanus	MW712963	Rm1	Vietnam	Ngoc Lac	V7	20.0944	105.3797	This study	
R. malayanus	MW712964	Rm1	Vietnam	Huong Hoa	V9	16.9370	106.5810	This study	

R. malayanus	MW712965	Rm2	Vietnam	Dakrong	V10	16.6000	106.8667	This study	
R. malayanus	HM541604	Rm20	Vietnam	Phu Quoc	V18	10.2000	104.0000	[1]	
R. shameli	MW712972-MW712981	Rsh1	Cambodia	Preah Vihear Temple	C1	14.3594	104.7859	This study	20 points, 30 points
R. shameli	MW712982, MW712983	Rsh1	Cambodia	Krong Preah Vihear	C2	13.7641	104.8592	This study	20 points, 30 points
R. shameli	MW712984-MW713001	Rsh1	Cambodia	SroLav	C3	13.7914	105.6022	This study	4 points, 20 points, 30 points
R. shameli	MW713002-MW713030	Rsh1	Cambodia	Virachey	C5	14.2032	107.0050	This study	20 points, 30 points
R. shameli	MW713031	Rsh2	Cambodia	Preah Vihear Temple	C1	14.3594	104.7859	This study	30 points
R. shameli	MW713032	Rsh3	Cambodia	Krong Preah Vihear	C2	13.7641	104.8592	This study	30 points
R. shameli	HM541778	Rsh4	Cambodia	KaohKong	C7	11.6390	103.5740	[1]	
R. shameli	MW713033, MW713034	Rsh5	Cambodia	Kampot	C8	10.5597	104.2822	This study	
R. shameli	HM541782, HM541783, HM541784, HM541793	Rsh1	Laos	Dong Hua Sao	L10	14.8220	106.0600	[1]	20 points, 30 points
R. shameli	HM541785	Rsh1	Laos	Nam Kading	L4	18.8420	104.5830	[1]	20 points, 30 points
R. shameli	HM541786	Rsh1	Laos	Vientiane	L6	18.0830	102.6670	[1]	20 points, 30 points
R. shameli	HM541780, HM541781, HM541792, JF444101	Rsh1	Laos	near Hin Nam No	L8	17.5670	105.8330	[1], unpublished	20 points, 30 points
R. shameli	HM541788, HM541789, HM541790, HM541791	Rsh1	Laos	Attapeu	L9	14.9500	107.1330	[1]	20 points, 30 points
R. shameli	HM541787, HM541794	Rsh6	Laos	Dong Kanthung	L11	14.1750	105.5750	[1]	30 points
R. shameli	HM541779	Rsh7	Laos	near Hin Nam No	L8	17.5670	105.8330	[1]	30 points
R. shameli	HM541777	Rsh5	Vietnam	PhuQuoc	V18	10.2000	104.0000	[1]	

References

1. Francis, C.M. *et al.* The role of DNA barcodes in understanding and conservation of mammal diversity in Southeast Asia. *PLoS ONE* **5**, E12575; doi:10.1371/journal.pone.0012575 (2010).
2. Wacharapluesadee, S. *et al.* Evidence for SARS-CoV-2 related coronaviruses circulating in bats and pangolins in Southeast Asia. *Nat. Commun.* **12**, 972; doi:10.1038/s41467-021-21240-1 (2021).
3. Zhou, P. *et al.* A pneumonia outbreak associated with a new coronavirus of probable bat origin. *Nature* **579**, 270-273; doi:10.1038/s41586-020-2012-7 (2020).
4. Ith, S. *et al*. Geographical Variation of *Rhinolophus affinis* (Chiroptera: Rhinolophidae) in the Sundaic Subregion of Southeast Asia, including the Malay Peninsula, Borneo and Sumatra. *Acta Chiropterol*. **18**, 141–161; doi:10.3161/15081109ACC2016.18.1.006 (2016).
5. Arai, S. *et al*. Molecular Phylogeny of Mobatviruses (Hantaviridae) in Myanmar and Vietnam. *Viruses* **11**, 228; doi:10.3390/v11030228 (2019).
6. Ith, S. *et al.* Taxonomic implications of geographical variation in *Rhinolophus affinis* (Chiroptera: Rhinolophidae) in mainland Southeast Asia. *Zool. Stud.* **54**, e31; doi:10.1186/s40555-015-0109-8 (2015).
7. Liang, J.; He, X.; Peng, X.; Xie, H.; Zhang, L. First record of existence of *Rhinolophus malayanus* (Chiroptera, Rhinolophidae) in China. *Mammalia* **84**, 362-365; doi:10.1515/mammalia-2019-0062 (2020).
8. Zhou, H. *et al*. A novel bat coronavirus closely related to SARS-CoV-2 contains natural insertions at the S1/S2 cleavage site of the spike protein. *Curr. Biol*. **30**, 3896; doi:10.1016/j.cub.2020.09.030 (2020).
9. Soisook, P *et al*. Hill forest dweller: A new cryptic species of *Rhinolophus* in the '*pusillus* group' (Chiroptera: Rhinolophidae) from Thailand and Lao PDR. *Acta Chiropt.* **18**, 117-139; doi:10.3161/15081109ACC2016.18.1.005 (2016).

Table S2. Geographic records used to infer the ecological niche of bat viruses related to SARS-CoV.

Country	locality	Latitude	Longitude	References
China	Guangdong	22.65334	113.05734	[1,2]
China	Guangxi, Nanning	22.81700	108.36653	[1,3]
China	Guizhou, Anlong	25.09900	105.44269	[4]
China	Hebei	38.71913	114.80067	[1,2]
China	Henan, Jiyuan	35.06725	112.60192	[1,4]
China	Hong Kong	22.37425	114.08477	[5]
China	Hong Kong	22.42819	114.06778	[5]
China	Hubei, Yichang	30.69197	111.28647	[3]
China	Jilin, Tonghua county	41.67981	125.75925	[1,6]
China	Shaanxi	33.99589	107.48343	[1,2]
China	Shanxi	37.27425	111.24516	[1,2]
China	Yunnan, Baoshan	25.11206	99.16175	[7]
China	Yunnan, Kunming	24.88008	102.83289	[7-9]
China	Yunnan, Lufeng	25.15011	102.07903	[10]
China	Yunnan, Mangshi	24.43369	98.58808	[11]
China	Yunnan, Menglian	22.32911	99.58417	[6]
China	Zhejiang, Longquan	28.07464	119.14147	[4]
China	Zhejiang, Daishan	30.26671	122.22394	[1,12]
Korea	Jeongbuk	35.42772	127.13625	[13,14]

References

1. Wu, Z. *et al*. Deciphering the bat virome catalog to better understand the ecological diversity of bat viruses and the bat origin of emerging infectious diseases. *The ISME journal* **10**, 609-620; doi:10.1038/ismej.2015.138 (2016).
2. Fan, Y.; Zhao, K.; Shi, Z. L.; Zhou, P. Bat coronaviruses in China. *Viruses* **11**, 210; doi:10.3390/v11030210 (2019).
3. Li, W. *et al*. Bats are natural reservoirs of SARS-like coronaviruses. *Science* **310**, 676-679; doi:10.1126/science.1118391 (2005).
4. Lin, X.D. *et al*. Extensive diversity of coronaviruses in bats from China. *Virology* **507**, 1-10; doi:10.1016/j.virol.2017.03.019 (2017).
5. Lau, S.K. *et al.* Severe acute respiratory syndrome coronavirus-like virus in Chinese horseshoe bats. *Proc. Natl. Acad. Sci. U.S.A.* **102**, 14040-14045; doi:10.1073/pnas.0506735102 (2005).
6. Xu, L. *et al*. Detection and characterization of diverse alpha- and betacoronaviruses from bats in China. *Virol. Sin.* **31**, 69-77; doi:10.1007/s12250-016-3727-3 (2016).
7. Ge, X.Y. *et al*. Isolation and characterization of a bat SARS-like coronavirus that uses the ACE2 receptor. *Nature* **503**, 535-538; doi:10.1038/nature12711 (2013).
8. He, B. *et al*. Identification of diverse alphacoronaviruses and genomic characterization of a novel severe acute respiratory syndrome-like coronavirus from bats in China. *J. Virol.* **88**, 7070-7082; doi:10.1128/JVI.00631-14 (2014).
9. Hu, B. *et al*. Discovery of a rich gene pool of bat SARS-related coronaviruses provides new insights into the origin of SARS coronavirus. *PLoS Pathog.* **13**, e1006698; doi:10.1371/journal.ppat.1006698 (2017).

10. Lau, S.K. *et al*. Severe acute respiratory syndrome (SARS) coronavirus ORF8 protein is acquired from SARS-related coronavirus from greater horseshoe bats through recombination. *J. Virol.* **89**, 10532-10547; doi:10.1128/JVI.01048-15 (2015).
11. Wang, L. *et al*. Discovery and genetic analysis of novel coronaviruses in least horseshoe bats in southwestern China. *Emerg. microbes & infect.* **6**, e14; doi:10.1038/emi.2016.140 (2017).
12. Hu, D. *et al*. Genomic characterization and infectivity of a novel SARS-like coronavirus in Chinese bats. *Emerg. microbes & infect.* **7**, 154; doi:10.1038/s41426-018-0155-5 (2018).
13. Lee, S. *et al.* Genetic characteristics of coronaviruses from Korean bats in 2016. *Microb. Ecol.* **75**, 174-182; doi:10.1007/s00248-017-1033-8 (2018).
14. Kim, Y. *et al*. Complete genome analysis of a SARS-like bat coronavirus identified in the Republic of Korea. *Virus genes* **55**, 545-549; doi:10.1007/s11262-019-01668-w (2019).

Supplementary Figure S1. Model performance in geographic space on training data. Ecological niche of bat viruses related to SARS-CoV (SCoVrCs).

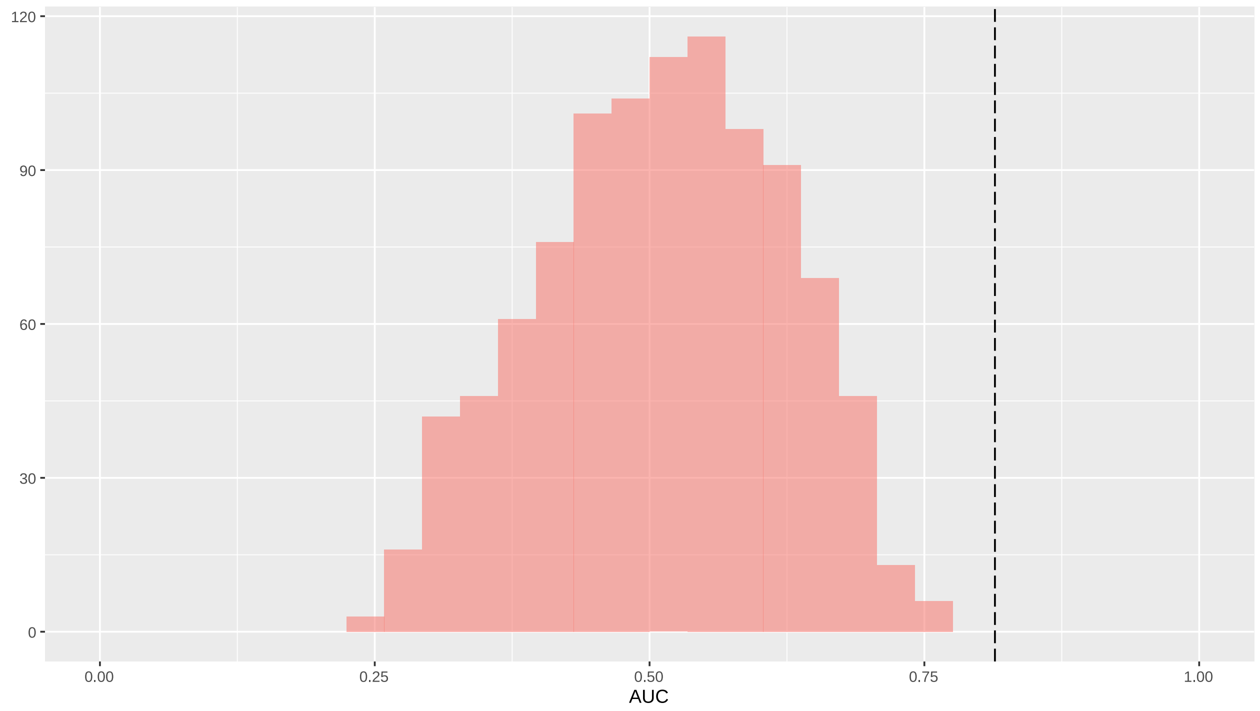

Supplementary Figure S2. Model performance in geographic space on training data. Ecological niches of bat viruses closely related to SARS-CoV-2 (SCoV2rCs) predicted using 4 points (data set A).

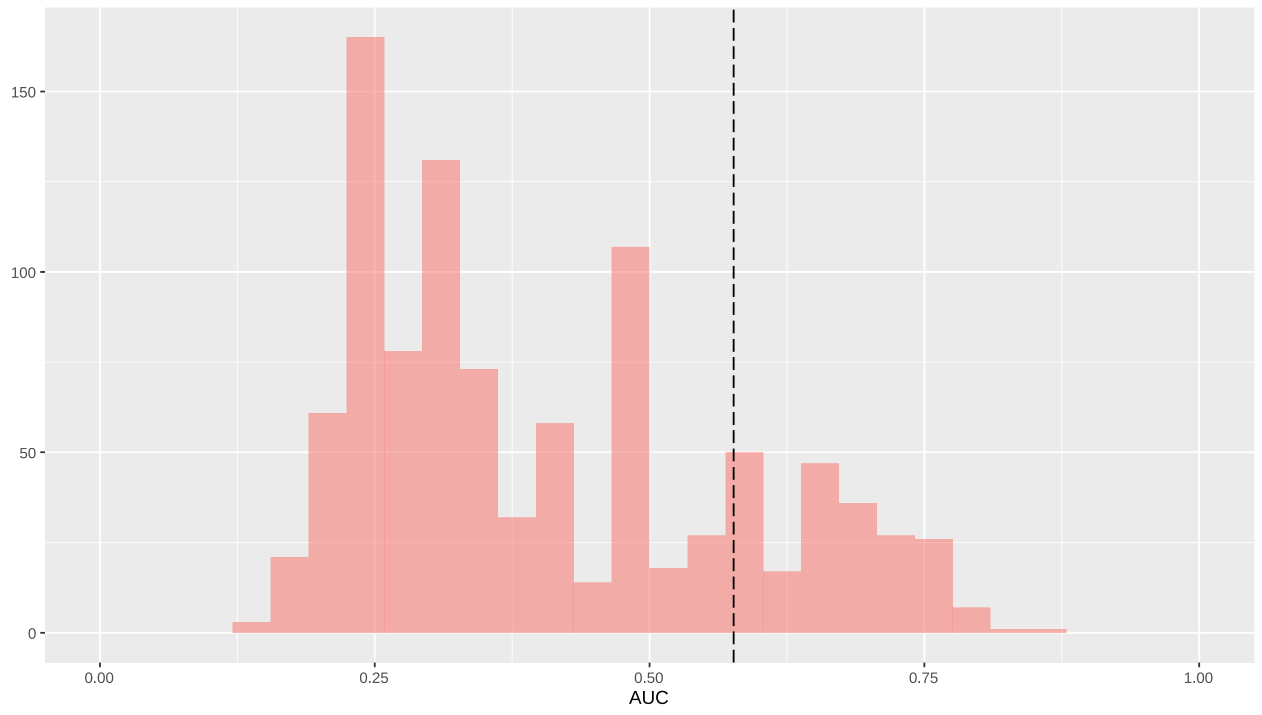

Supplementary Figure S3. Model performance in geographic space on training data. Ecological niches of bat viruses closely related to SARS-CoV-2 (SCoV2rCs) predicted using 21 points (data set B).

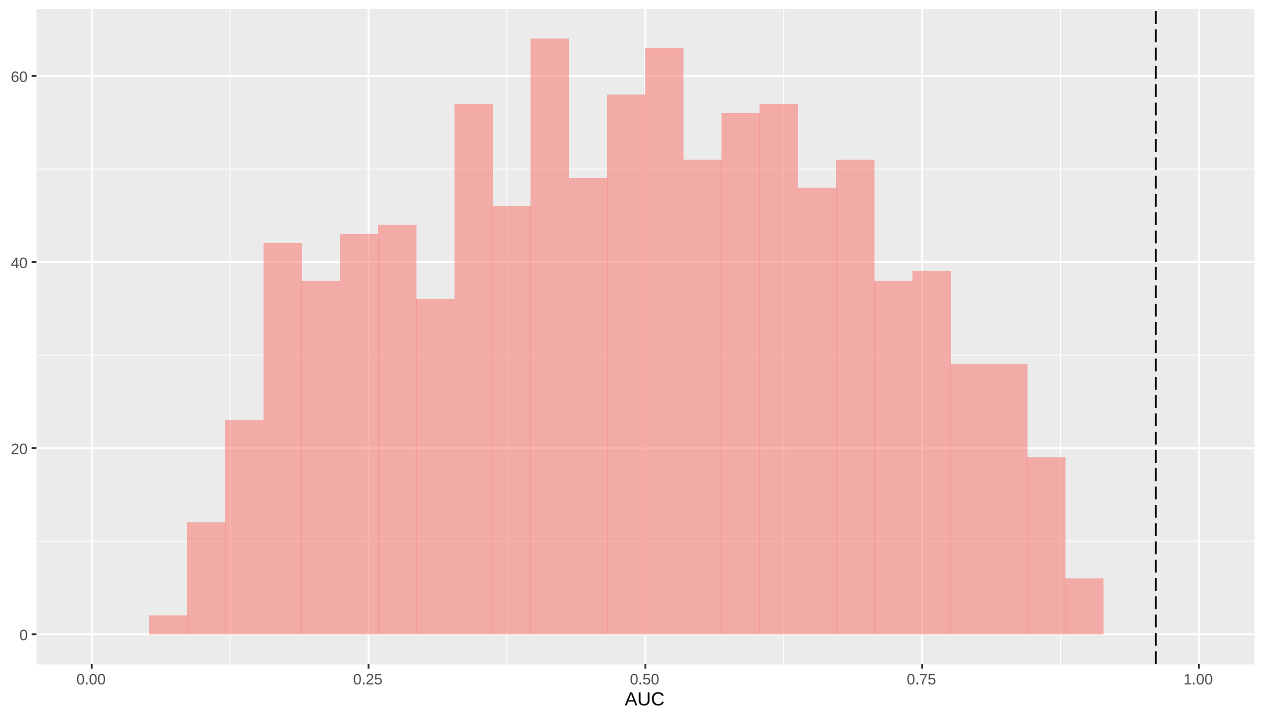

Supplement: Supplementary file 1 — Supplementary Information. [file 41598_2021_93738_MOESM1_ESM.pdf]
